# Cheap, Gram-Scale Fabrication of BN Nanosheets via Substitution Reaction of Graphite Powders and Their Use for Mechanical Reinforcement of Polymers

**DOI:** 10.1038/srep04211

**Published:** 2014-02-27

**Authors:** Fei Liu, Xiaoshu Mo, Haibo Gan, Tongyi Guo, Xuebin Wang, Bin Chen, Jun Chen, Shaozhi Deng, Ningsheng Xu, Takashi Sekiguchi, Dmitri Golberg, Yoshio Bando

**Affiliations:** 1 State Key Laboratory of Optoelectronic Materials and Technologies, Guangdong Province Key Laboratory of Display Material and Technology, and School of Physics and Engineering, Sun Yat-sen University, Guangzhou 510275 (PR China); 2 Inorganic Nanostructured Materials Group, World Premier International (WPI) Center for Materials Nanoarchitectonics (MANA), National Institute for Materials Science (NIMS), Namiki 1-1, Tsukuba, Ibaraki, Japan 305-0044; 3 Nano-Electronics Materials Unit, World Premier International (WPI) Center for Materials Nanoarchitectonics (MANA), National Institute for Materials Science (NIMS), Namiki 1-1, Tsukuba, Ibaraki, Japan 305-0044; 4 Nanotube Group, World Premier International (WPI) Center for Materials Nanoarchitectonics (MANA), National Institute for Materials Science (NIMS), Namiki 1-1, Tsukuba, Ibaraki, Japan 305-0044

## Abstract

As one of the most important two-dimensional (2D) materials, BN nanosheets attracted intensive interest in the past decade. Although there are many methods suitable for the preparation of BN sheets, finding a cheap and nontoxic way for their mass and high-quality production is still a challenge. Here we provide a highly effective and cheap way to synthesize gram-scale-level well-structured BN nanosheets from many common graphite products as source materials. Single-crystalline multi-layered BN sheets have a mean lateral size of several hundred nanometers and a thickness ranging from 5 nm to 40 nm. Cathodoluminescence (CL) analysis shows that the structures exhibit a near band-edge emission and a broad emission band from 300 nm to 500 nm. Utilization of nanosheets for the reinforcement of polymers revealed that the Young's modulus of BN/PMMA composite had increased to 1.56 GPa when the BN's fraction was only 2 wt.%, thus demonstrating a 20% gain compared to a blank PMMA film. It suggests that the BN nanosheet is an ideal mechanical reinforcing material for polymers. In addition, this easy and nontoxic substitution method may provide a universal route towards high yields of other 2D materials.

In recent years, various two-dimensional (2D) nanomaterials have received considerable developments being driven by numerous intriguing results on graphene^1^,^2^,^3^,^4^,^5^,^6^,^7^,^8^. Among them, BN products have attracted more and more attention because of their high elastic modulus, high melting-point, superb thermal conductivity and a large and direct band gap. Such properties can be of high value for ultraviolet-light emitters, advanced ceramic composites, electrical insulators, solid lubricants and ideal substrates for graphene electronics^5^,^9^,^10^,^11^,^12^,^13^,^14^,^15^,^16^,^17^,^18^. Although much effort has been devoted to the preparation of 2D BN sheets^15^,^16^,^19^,^20^,^21^,^22^,^23^,^24^,^25^,^26^,^27^,^28^,^29^,^30^,^31^,^32^,^33^,^34^,^35^,^36^, it is still a challenge to effectively fabricate single-crystalline uniformly structured 2D BN sheets with high yield at low cost and without using toxic reagents.

Chemical vapor deposition (CVD) is a low-cost well controllable process for the mass production of nanomaterials. Among various CVD methods, the so-called “substitution route” is of particular interest for nano-BN production because BN nanosheets have a geometrical configuration similar to graphene (sp^2^ hybridization). Thus this technique has been thought to be one of the most effective methods for the growth of BN or BCN nanotubes^37^,^38^,^39^,^40^. But to the best of our knowledge, only two research groups have applied the substitution reactions to prepare BN or BCN sheets^35^,^36^. For example, Han et al. used a two-step way and utilized boron oxide and metal oxide powders, and graphene sheets as source materials kept at 1650°C in nitrogen atmosphere for 30 min. The product was collected from the surface of graphene sheets and treated in air at 600–650°C for 30 min^35^. In Lin's report, a three-step reaction was applied to synthesize BCN nanosheets^36^. Mono-layered graphene oxide (GO) nanosheets were firstly fabricated by a modified Hummer's method^41^. Secondly, mono-layered GO was deposited on the surface of a Si substrate by immersing the substrate into the GO nanosheet solution. Finally, the GO nanosheets and B_2_O_3_ powders were used to fabricate BCN nanosheets at 900–1100°C in Ar/NH_3_ atmosphere, and the product was collected from the Si substrate surface after the growth over 30 min. Compared with these two existing substitution methods, the proposed synthesis method by us has the following advantages. It is one-step route, which leads to highly-crystalline products and does not need expensive and/or toxic precursors. In addition, the mass of the BN product may reach grams and its yield is 25% with respect to the starting materials. These features are beneficial for their future wide-spread applications.

In this paper, several graphite sources have been tried to synthesize 2D BN sheets at gram levels. The growth mechanism has also been discussed. Finally, CL measurements and the experiments aimed at the reinforcement of polymers have been performed.

## Discussion

**Figure 1:**
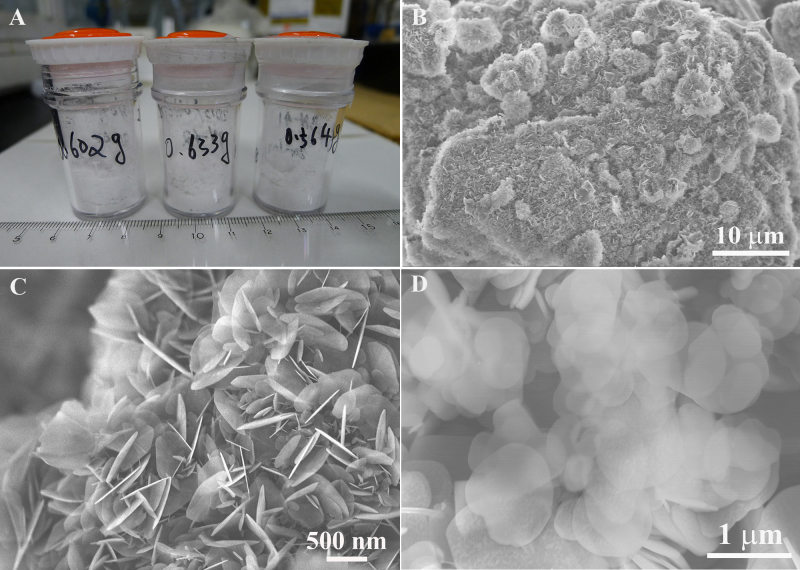
(A) A photograph of the BN sheets prepared in gram scale. (B, C) Low- and high-magnification SEM images of the BN sheets. (D) Top view of the BN sheets.

By controlling the growth conditions, gram-level BN sheets have been successfully fabricated using different graphite sources. The morphology and the yield of the “snow-white” BN products are very similar independent of graphite sources used, as seen in Table 1. Typical photographs of the BN products are presented in Fig. 1A. BN products mass ranges from 0.6 g to 1.1 g per experimental run, and their corresponding yield (with respect to a mass of the starting precursors) ranges between 15% and 26%. Because the morphology and chemical compositions of the BN products using different graphite sources were very close, here we selectively present the results peculiar to a graphite micropowder source. Low- and high-magnification SEM images of the BN products are respectively shown in Figs. 1B and 1C. The products are very uniform. The underneath sheets are clearly seen through the top sheets. The BN sheets have a polygonal appearance and smooth surfaces (Figure 1D), and their lateral dimensions range from 300 nm to 800 nm. All regarded sheets were prepared in pure N_2_ atmosphere using cheap and nontoxic graphite sources, as seen in Table 1.

The possible formation mechanism of BN sheets is thought to be as follows. Firstly, B_2_O_3_ solid powders transform into the B_2_O_2_ vapor at a high temperature, over 1200°C, as evidenced by massive white smog flowing through the chamber in all experiments. The reaction equation can be written as: 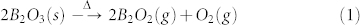
This is consistent with the experimental results from other research groups^35^,^48^. Secondly, oxygen broke carbon atom bonds starting from the defect sites of their honeycomb lattice. Thirdly, B_2_O_2_ vapors reacted with the activated carbon atoms (with those having broken bonds) and gradually replaced their original lattice sites under the continuous high-temperature substitution reaction. Lastly, once the high-temperature reaction was over, all former carbon sites within the graphite's honeycomb lattice were filled with alternating B and N atoms. This step can be expressed as: 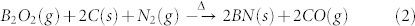
If the growth time is not long enough, e.g. 1–2 h, some C contents can still be detected by high-resolution transmission electron microscopy (HRTEM) and electron energy loss spectroscopy (EELS) techniques. It is attributed to the incomplete carbon substitution reaction, as illustrated in other works^35^,^48^,^49^. The position of the source materials in the crucible was found to be crucial for the formation of pure BN sheets. Usually, B_2_O_3_ powders were placed beneath the graphite powders because B_2_O_2_ vapors have to have enough time to react with the precursors. The suitable source materials' ratio is also very important for the present synthesis. If the ratio of graphite powders to the B_2_O_3_ powders is too high or low, BCN/BN complexes may be found in the products rather than pure BN sheets. Therefore, based on the aforementioned experimental results and analysis, the C-substitution reaction should be responsible for the BN sheet's formation, this resembles a transition from carbon nanotubes (CNTs) to BN nanotubes documented in other studies^35^,^49^. More detailed discussions on the effect of the growth conditions on the morphology and the compositions of the products can be found in Supporting Information.

**Figure 2:**
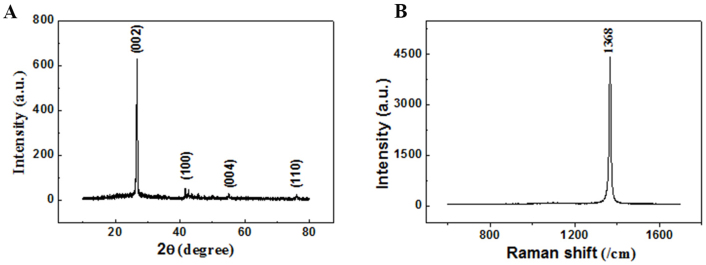
(A) Typical XRD pattern of the BN sheets. (B) Corresponding Raman spectrum.

XRD technique was firstly used to analyze the as-grown sheets. Typical XRD pattern of the product is given in Fig. 2A. There are only a strong h-BN (002) peak and three very weak peaks (corresponding to (100), (004) and (100) h-BN planes) in the pattern. Thus the product can be considered to be pure highly-crystalline hexagonal BN phase. Raman spectrum of the product is shown in Fig. 2B. Only an intense and sharp peak at 1368 cm^−1^ can be found in the spectrum, which is close to the characteristic Raman peak of bulk BN materials^42^,^43^,^44^. This peak should be attributed to the high frequency intralayer E_2g_ vibration mode of h-BN, which is in a good agreement with other reports^21^,^42^,^43^,^44^,^45^,^46^,^47^. FWHM of the present characteristic peak is only 10 cm^−1^, which is narrower than that in the former reports (13 cm^−1^ 45, 16 cm^−1^ 44, 19 cm^−1^ 21, 27 cm^−1^ 46 and 30 cm^−1^ 47). It suggests that the BN sheets by the present substitution reaction have better crystalline quality.

**Figure 3:**
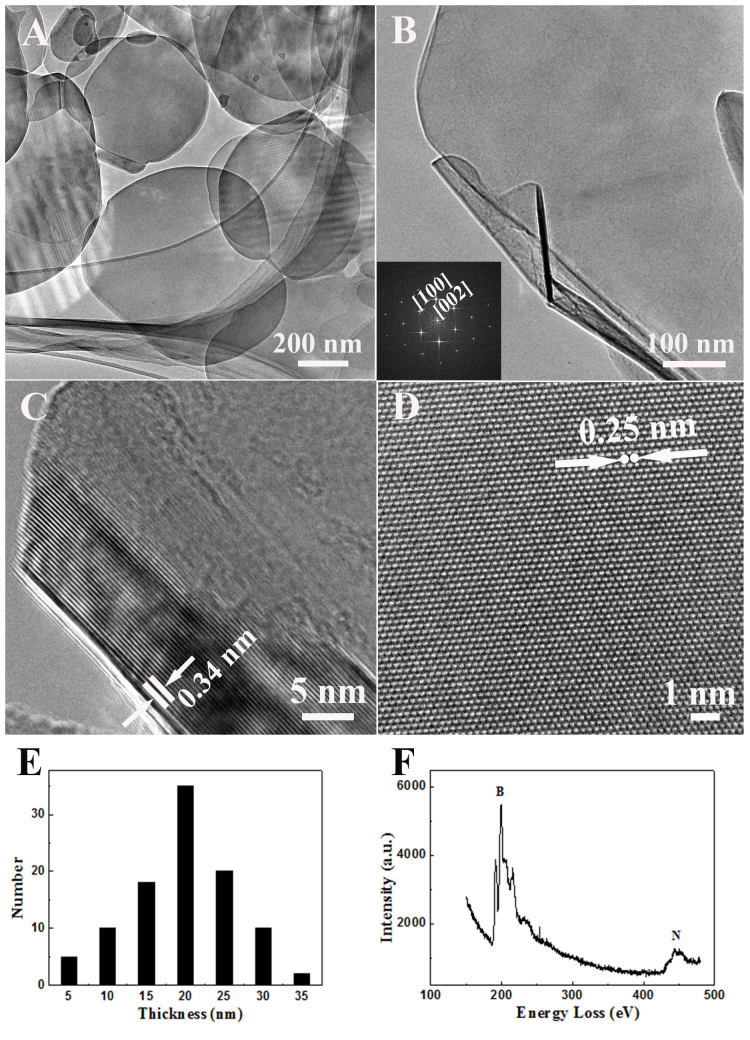
(A, B) Low resolution TEM images of the BN sheets at different magnifications. The inset is the SAED pattern. (C, D) High resolution TEM images of the edge and the central regions of the BN sheet. (E) Statistics diagram of the BN sheets' thickness distribution. (F) Typical EEL spectrum of the BN sheet.

Figures 3A and 3B respectively give the low-magnification and high-magnification TEM images of BN sheets. Their surface is rather smooth, in agreement with the SEM results in Fig. 1C. The corresponding SAED pattern is sharp and clear (inset), which proves that the sheets are perfect single crystals. The nanosheet in Fig. 3C consists of 15–20 layers and the mean spacing between adjacent layers is ~0.33–0.34 nm, which corresponds to the lattice spacing of graphitic (002) plane. The atomically-resolved TEM image of the nanosheet is shown in Fig. 3D. A honeycomb atomic lattice peculiar to a graphitic structure is apparent. A distance between each two nearest atoms marked by the white dots is 0.25 nm, which matches the well-known B-B or N-N atom distance in the (100) plane of h-BN lattice^15^,^16^,^27^. Based on the statistic results on the lateral thickness of the sheets during TEM measurements, the most common sheets' thickness was found to range from 5 nm to 35 nm in Fig. 3E. The average number of layers is ~60; this number can be further reduced to 10–15 after applying high-power sonication and high-speed centrifugation, as discussed in our earlier studies^15^,^16^. EELS analysis (Figure 3F) also indicates the pure BN sheet composition. Combining all data together, it is proved that pure h-BN single crystals were indeed fabricated.

**Figure 4:**
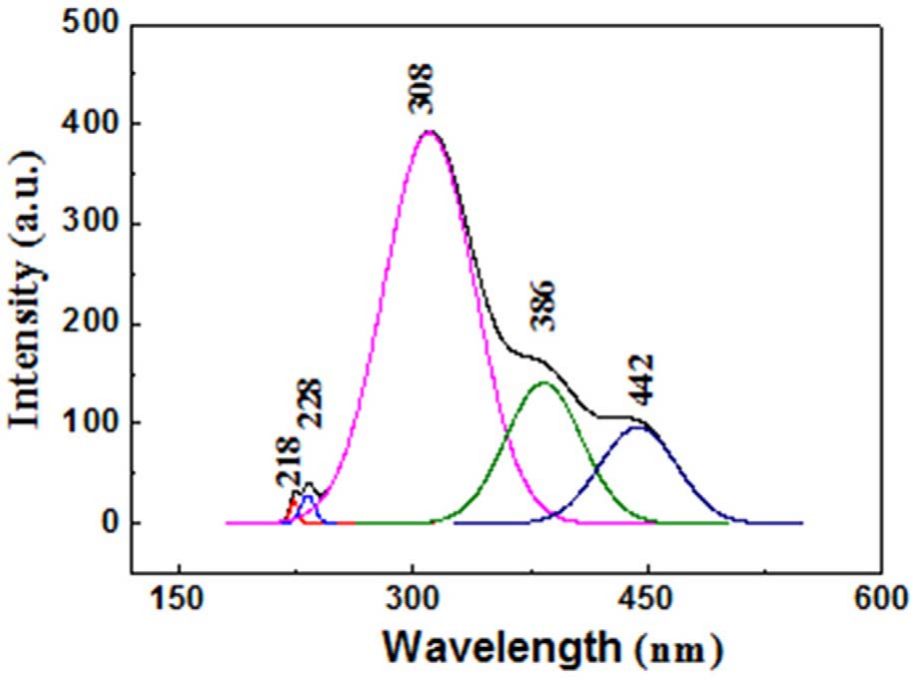
Representative CL spectrum of the BN sheets.

To understand the intrinsic optical properties of the BN sheets as well as further study their crystallinity, room-temperature CL measurements using a 5 keV accelerating voltage were performed on the sample. Representative CL spectrum of the BN sheets is given in Fig. 4, in which one can find it is deconvoluted into 5 peaks. The two peaks at 218 nm and 228 nm represent the near band-edge emission. They are usually assigned to the intrinsic excitonic recombinations in BN materials^21^,^27^,^50^,^51^,^52^,^53^, corresponding to their band gap (~5.7 eV). Other three broad emission peaks are distributing from 300 nm to 500 nm, which respectively locate at 308 nm (4.03 eV), 386 nm (3.22 eV) and 442 nm (2.81 eV). These three characteristic peaks may originate from B vacancies and residual C or O impurities^21^,^27^,^50^,^51^,^52^,^53^.

**Figure 5:**
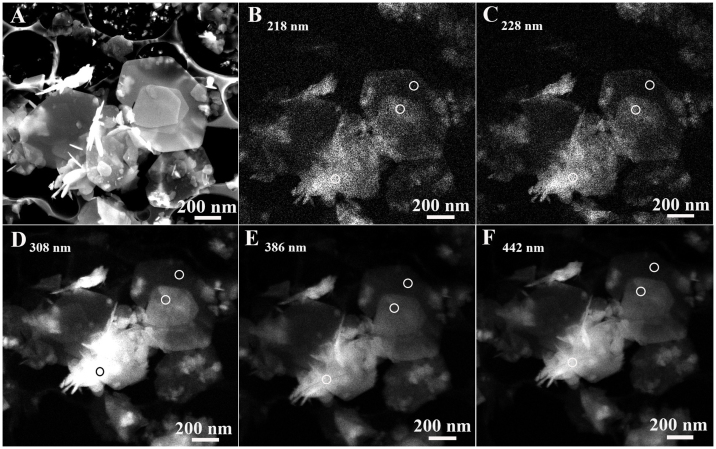
(A) Polychromatic CL image of the BN sheets. (B–F) Monochromatic CL images of the BN sheets at 218 nm, 228 nm, 308 nm, 386 nm and 442 nm wavelength illuminations. From the right to the left, three regions referred by the white circles are respectively named as region 1, region 2 and region 3.

Subsequently, the spatial distribution images corresponding to the BN sheet's CL spectrum are demonstrated in Figure 5 to explain the origin of the emission peaks. Figure 5A gives the panchromatic CL image of the BN sheets, in which one can see that the sheets exhibit homogeneous emission intensity on their planar surfaces. The thick regions such as the edges of vertically-standing sheets and the regions of overlapped sheets exhibit higher brightness than thin regions. The monochromatic CL images taken at different wavelengths of 218 nm, 228 nm, 308 nm, 386 nm and 442 nm are shown in Figs. 5B–5F, respectively. By comparing the CL images of the sheets at 218 nm and that at 228 nm, it is found that the brightness of all the sheets in the images is rather close except some thick regions. To better understand the CL properties, the same three regions with different thickness were chosen to investigate the luminescent intensity, as marked by three white circles in their CL images. The relative intensity can be quantitatively analyzed and compared because the brightness reflects the luminescent intensity, as referred by the inset images of Fig. 5. Based on this method, their brightness ratio of region 1 to region 2 is calculated to be ~1:3, meanwhile the brightness ratio of region 2 to region 3 is ~3:5 under 218 nm or 228 nm illuminations. But when the CL mapping was respectively taken at 308, 386 and 442 nm, the mean luminescent intensity ratio of region 1 to region 2 changes to ~1:6 and the average luminescent intensity of region 2 to region 3 changes to ~1:5. These discrepancies may come from different defect or impurity density of the BN sheets. In fact, Region 1 is the thinnest region of the BN sheet, and region 2 is relatively thicker region composed of two overlapped sheets. Because the luminescence of the BN sheets at 218 nm or 228 nm originates from the near band-edge emission of BN sheet, thicker region 2 corresponding to more BN layers should exhibit higher CL intensity, as indeed observed in CL images. In comparison with regions 1 and 2, region 3 is the lateral region of BN sheets which has the largest thickness. It is nearly vertical to the planar regions 1 and 2 in Fig. 5A. Therefore, it can be understood that the standing sheets (region 3) exhibit the highest CL intensity. For the luminescent behaviors of BN sheets at 308 nm, 386 nm and 442 nm, there is some difference from those at 218 and 228 nm because the luminescence at these wavelengths may result from B vacancies, residual C or O impurities according to the aforementioned interpretations^21^,^27^,^50^,^51^,^52^,^53^. It is noted that a slight variation in the defect or impurity's density should lead to a large difference of the luminescence. Usually, most of the impurities or defects exist at sheet edges. As a result, region 3 of the sheets should have a higher density of defects or impurities compared to other regions which leads to the highest luminescent intensity in CL images. Likewise, it is reasonable that the luminescence intensity ratio of region 2 to region 1, or region 3, or region 2 at the wavelengths of 308 nm, 386 nm and 442 nm is higher than that at 218 nm or 228 nm. Based on the CL results, because obvious near band-edge emission of the sheets has been observed in CL and other peaks are only related to a few point defects or impurities, it further reveals the sheets have high crystallinity. And their high crystallinity suggests that they may have better mechanical performances.

**Figure 6:**
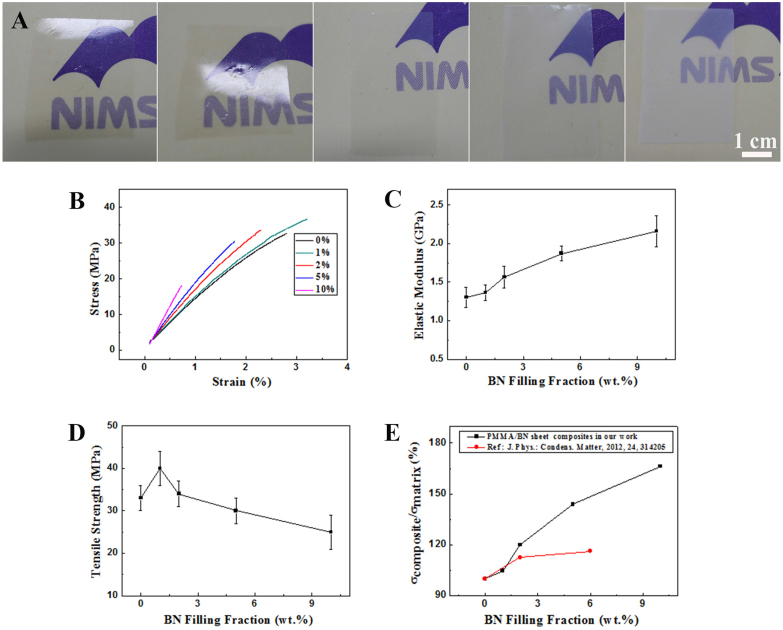
(A) Photographs of the BN/PMMA composite film at (a) 0 wt.% BN; (b) 1 wt.% BN; (c) 2 wt. % BN; (d) 5 wt.% BN; (e) 10 wt.% BN. (B) Stress-strain curves of the BN/PMMA composite films with different BN filling fractions. (C, D) Elastic modulus versus BN filling fraction curve and tensile strength versus BN filling fraction curve of the composite film, respectively. (E) The curves of the modulus reinforcing ratio of the composite film to the BN filling fraction for the BN sheets prepared by different methods.

At last, we prepared BN-nanosheet/polymethyl methacrylate (PMMA) composites to study the possible reinforcement for a polymer matrix. The as-grown BN sheets and PMMA were uniformly dispersed into the dimethylformamide (DMF) solutions and mixed together, then cast to form a thin film. The films were dried at 80°C; more detailed fabrication process can be found in the Experimental Section. The photographs of BN/PMMA composite films at different BN fractions (0 wt.%, 1 wt.%, 2 wt.%, 5 wt.%, 10 wt.%) are shown in Fig. 6A. Although the transparency of films gradually declines with an increase in BN sheet's fraction, they still exhibit decent transparency even at 10 wt.% loading fraction. The detailed optical transparency data can be found in Fig. S2 (Supporting Information). When BN fraction in the composite is smaller than 5 wt.%, the transparency is still more than 66%. A typical stress-strain curve of the composite film is given in Fig. 6B. Because the linear relation between the tensile stress and the strain can be clearly seen in the curve, the tensile behaviors of the BN-nanosheet/PMMA composite film can be determined to obey the classical Hook's law. The elastic modulus of the PMMA is found to increase from 1.30 GPa to 2.16 GPa when the filling fraction of the BN sheets becomes 10 wt.%. The tensile strength-BN filling fraction and elastic modulus-BN filling fraction curves are respectively given in Figs. 6C and 6D. The elastic modulus of PMMA has enhanced to 1.56 GPa when the BN's fraction is only 2 wt.%, this corresponds to a 20% increase compared to a blank PMMA. Particularly, one can see in Figure 6C that the film's modulus can still continue to increase with an increase of the BN's fraction and doesn't arrive at the climax within our measurement range. The composite film at 1 wt.% BN filling fraction possesses the highest tensile strength (40 MPa), i.e., an increase by 21.2% with respect to the blank PMMA (33 MPa). When the BN filling fraction is 2 wt.%, the tensile strength of the film is 34 MPa, which is slightly higher than that of the blank PMMA. If BN sheet fraction further increases, the tensile strength of the composite film will deteriorate, as seen in Fig. 6D. Therefore, 2 wt.% of the BN sheet loading fraction is thought to be the optimal value based on the considerations of the optical transparency, elastic and tensile properties. Further comparison on the reinforcement effect was made between the present BN sheets and those produced previously, as shown in Fig. 6E. In the curves, σ_matrix_ and σ_composite_ are respectively the elastic modulus of the blank PMMA film and the composite film. As found in Fig. 6E, the Young's modulus of the composite film produced via the present route increases by 20% when the BN's fraction is 2 wt.%, which is higher than that measured before (14%). Also the reinforcement effect (43.8%) at 5 wt.% BN fraction is also better than that (35%) for a composite with a 6 wt.% BN fraction produced by us previously^27^. Such good performance may result from the high crystallinity and good dispersion of the sheets within the PMMA matrix. These results are also comparable with the good reinforcement effects of BN or C nanotubes in polymers^16^,^54^,^55^,^56^, which implies that the present BN nanosheets is a decent reinforcement materials for the polymer matrix composites.

By utilizing different graphite source materials, we have successfully realized cheap and non-toxic mass production of BN sheets with high purity and crystallinity. The mass and yield of the as-grown product can reach gram-scale and over 20%, respectively, which may be beneficial for their large-scale applications. BN sheets not only exhibit intrinsic UV emission but also have a broad emission due to a few defects or impurities. The elastic modulus of the BN/PMMA film can increase by 20% (up to 2.16 GPa) when the BN's fraction is only 2 wt.%, while large tensile strength and high transparency can simultaneously be maintained for this composite. We envisage that this easy and nontoxic substitution method may provide a universal route towards high yields of other 2D materials.

## Methods

### Synthesis method

Graphite micropowders (99% purity, <20 μm, Aldrich), graphite rod powders (99% purity, Aldrich), carbon nanotubes (>95% purity, Aldrich), activated carbon powders (99% purity, Aldrich) and amorphous carbon nanopowders (99% purity, Aldrich) were used as carbon sources. The B_2_O_3_ powders (99% purity, Wako) were used as boron source and loaded at the bottom of the BN crucible. The mass ratio of the source materials using different carbon sources is listed in Table 1. The carbon sources were put into the crucible to cover the surface of the B_2_O_3_ powders, which is essential to ensure the effective proceeding of the following substitution reaction. No catalysts were used. Then the crucible was placed into the central region of an induction furnace, as described in our previous work^15^. The base pressure of the vacuum chamber was lower than 10 Pa and the reaction pressure was kept ambient. The furnace was heated to 1300–1400°C in N_2_ (1–4 SLM) gas in 20–30 min. After the growth for 4–6 hours, the furnace was cooled to room temperature in N_2_ gas and “snow-white” BN nanosheet products were collected from the BN crucible.

### Morphology and structure characterization

A field-emission type scanning electron microscope (SEM, S-4800, Hitachi Corp.) and transmission electron microscope (TEM, JEM-3000F, JEOL Corp.) were used to investigate the morphology and crystalline structure of the as-prepared BN sheets, respectively. XRD (RINT 2000, Cu Kα radiation) and Raman spectroscopy (Ar-He ion laser at 514 nm, Spectra-Physics Beamlok 2060-RS laser) were used to confirm their chemical compositions. The cathodoluminescence (CL) properties of the samples were investigated at room temperature in a thermal field emission electron microscope (TFE-SEM, S4200, Hitachi Corp.) equipped with a high resolution CL spectrometer.

### Composite preparation and tensile measurements

To fabricate polymer/BN composites, the as-synthesized BN sheets with a mass of 1–1.5 g were firstly dissolved into the dimethylformamide (DMF) solution (200 ml). Secondly, the solution was treated at 8000 rpm for 20 h in a tip-type ultrasonicator to better realize the dispersion of the BN sheets in DMF solution and get rid of the insoluble large particles. Thirdly, the DMF solution was rotary-evaporated to obtain high-concentration BN nanosheet solutions. Fourthly, the BN solutions were mixed with the polymethyl methacrylate (PMMA)/chloroform solutions with different weight ratios. Lastly, the mixture was spread over the glass plate and naturally volatilized at atmosphere followed by drying at 80°C for 10 h. Tensile tests on composites with different BN fractions were carried out on an EZ-S-100N machine, Shimadzu Corporation.

## Author Contributions

F.L. wrote the main manuscript. X.W. carried out the mechanical measurement of the sample. F.L., X.M., T.G. and H.G. prepared and characterized the BN nanosheets. B.C. and T.S. involved in the CL measurement of the sample. N.X., S.D., J.C., Y.B. and D.G. gave the helpful discussion and polished this paper.

## Supplementary Material

Supplementary Information Supporting Information

## Figures and Tables

**Table 1:** Table showing the growth conditions and morphology of BN sheets using different carbon sources

Source Materials	Growth temperature (°C)	Morphology	Size (nm)	Thickness (nm)	Product mass (g)	Product yield*
B_2_O_3_:graphite micropowders = 3.5 g:0.7 g	1300–1600	Uniform sheets	300–900	5–40	0.65–1.1	15–26%
B_2_O_3_:graphite rod powders = 3.5 g:0.7 g	1300–1600	Uniform sheets	1000–2000	20–80	0.65–1.1	15–26%
B_2_O_3_:activated carbon powders = 3.5 g:0.7 g	1300–1600	Uniform sheets	500–1000	10–50	0.65–1.1	15–26%
B_2_O_3_:amorphous carbon nanopowders = 3.5 g:0.7 g	1300–1600	Uniform sheets	300–800	5–30	0.65–1.1	15–26%
B_2_O_3_:MWCNTs** powders = 3.4 g:0.5 g	1300–1600	Uniform sheets	200–700	5–30	0.6–1.0	15–26%
B_2_O_3_:DWCNTs*** powders = 3.4 g:0.5 g	1300–1600	Uniform sheets	100–500	5–20	0.6–1.0	15–26%
B_2_O_3_:SWCNTs**** powders = 3.4 g:0.5 g	1300–1600	Uniform sheets	100–500	5–20	0.6–1.0	15–26%

*The product yield is calculated with respect to the staring precursors mass.

**Multi-walled carbon nanotubes.

***Double-walled carbon nanotubes.

****Single-walled carbon nanotubes.
