# Supplementary material for: Cheap, Gram-Scale Fabrication of BN Nanosheets via Substitution Reaction of Graphite Powders and Their Use for Mechanical Reinforcement of Polymers
Source: Sci Rep. 2014 Feb 27;4:4211. doi: 10.1038/srep04211 (PMC3936228; doi:10.1038/srep04211)
Supplement: Supplementary Information — Supporting Information [file srep04211-s1.doc]

***Supporting Information***

*(Submitted to* ***Scientific Reports****)*

**Cheap, Gram-Scale Fabrication of BN Nanosheets via Substitution Reaction of Graphite Powders and Their Use for Mechanical Reinforcement of Polymers**

Fei Liu1[[1]](#footnote-2)†, 2, Xiaoshu Mo^1^, Haibo Gan^1^, Tongyi Guo^1^, Xuebin Wang^2,†^, Bin Chen^3^, Jun Chen^1^, Shaozhi Deng^1^, Ningsheng Xu^1^,Takashi Sekiguchi^3^, Dmitri Golberg^4^,Yoshio Bando^2^

**Table of Contents**

Characterization of BN sheets by different graphitic sources……………………………… S3

The effect of the growth conditions on the product………………………………………… S4

Optical transparency of the BN/PMMA film with different BN fractions………………… S5

Figure and Table Captions……………………………………………………………………S6

Supplement references………………………………………………………………………S7

XRD patterns and Raman spectra of the BN sheets by different graphitic sources………… S8

SEM images of the BN sheets by different graphitic sources………………………………S9

**Characterization of the BN sheets by different graphitic sources**

Graphite micropowders, graphite rod powders, MWCNTs, DWCNTs, SWCNTs, activated C powders and amorphous C nanopowders have chosen to be the source materials for the synthesis of the BN sheets. The XRD patterns and the Raman spectra of the sheets grown by different graphite sources are given in Figs. S1A and S1B. From Figs S1A, only (002), (100), (101), (102), (004) and (100) peaks are seen in the XRD patterns, which are all indexed to belong to h-BN phase. Further investigation show that there is only a strong and sharp peak at 1368 cm^-1^ in the Raman spectra (Fig. S1B), which is proven to be the characteristic Raman peak of bulk BN materials [1-4]. So based on the XRD and Raman results, all the sheets are confirmed to be pure h-BN single crystals whatever kind of graphite powders were used as source materials.

**The effect of the growth conditions on the product**

By carrying lots of experiments, the effect of the growth conditions on the product is summarized as follows. Firstly, the growth temperature over 1300 ^o^C is necessary for the formation of pure BN sheets with high quality. When the growth temperature is lower than 1200 ^o^C, there are many big BN particles coexisting with the BN sheets and the product is confirmed to contain carbon contamination by Raman and EDX analysis. Secondly, the mass ratio of the B_2_O_3_ powders to the graphite powders is vital for the synthesis of BN sheets. Our research shows that the mass ratio of the B_2_O_3_ powders to the graphite powders should be kept ranging from 5:1 to 7:1, which is the most suitable ratio for the fabrication of single crystalline BN sheets. If the source ratio is too low or too high, there are usually many BN, BCN particles or unreacted graphite materials in the products. Thirdly, enough growth time is also needed for the formation of BN sheets. In general, snow-white BN sheets with high purity can be synthesized in the BN crucible when the growth time is over 4 hours. And if the growth time is lower than 4 hours, one can observe that there are some residual black graphite powders remaining in the crucible because the source materials have not enough time to be converted. Finally, the place position of the source materials is the crucial factor for mass production of pure BN sheets. If the B_2_O_3_ powders are mixed with the graphite powders or placed at the top of the graphite powders, the obtained products will be composed of BN, BCN sheets and the unreacted graphite powders. At this situation, the B_2_O_3_ vapor can’t effectively react with C before it goes through the chamber so that many graphitic powders or the BCN by-products are remained in the crucible. Therefore, to ensure the successful growth of high purity BN sheets, the B_2_O_3_ powders must be placed beneath the graphitic powders.

**Optical transparency of BN/PMMA film with different BN fractions**

The transmission properties of the BN/PMMA composite film with different BN fractions are compared to investigate their application potential. The smooth BN/PMMA films have a mean thickness of 20 μm. Their optical transparency curves are provided in Fig. S2. It is seen that the optical transparency of the composite film is higher than 66 % in the whole measurement range (300-800 nm) when the fraction of the BN sheets is 1 wt. % and 2 wt. %. It suggests that the composite films at such fractions have good optical transmission properties, which is in agreement with the observed results from the film photographs (Fig. 6A). It is also found that the transparency of the composite film will decrease with an increase in BN fraction. When the BN fraction increases to 10 wt. %, the transparency of the composite film decreases to 36 %. Moreover, it is clearly seen that there is the fast decline of the transparency curve with a decrease of the wavelength. It should be attributed to the existence of the BN fillers, which induce strong UV absorption because they have a wide direct band gap (5-6 eV), as mentioned by Zhi et al. [5].

**Supplemental References**

[1] Hoffman, D. M., Doll, G. L. & Eklund, P. C. Optical properties of pyrolytic boron nitride in the energy range 0.05—10 eV. *Phys. Rev. B* **30**, 6051-6056 (1984).

[2] Wu, J. et al. Raman spectroscopy and time-resolved photoluminescence of BN and B_x_C_y_N_z_ nanotubes. *Nano Lett.* **4**, 647-650 (2004).

[3] Saha, S. et al. Comparative high pressure Raman study of boron nitride nanotubes and hexagonal boron nitride. *Chem. Phys. Lett.* **421**, 86-90 (2006).

[4] Geick, R., Perry, C. H. & Rupprecht, G. Normal modes in hexagonal boron nitride. *Phys. Rev.* **146**, 543-547 (1966).

[5] Zhi, C. Y., Bando, Y., Tang, C. C., Kuwahara, H. & Golberg, D. Large-scale fabrication of boron nitride nanosheets and their utilization in polymeric composites with improved thermal and mechanical properties. *Adv. Mater.* **21**, 2889-2893 (2009).

**Table and Figure captions**

Fig. S1 XRD patterns and Raman spectra of the products using different graphitic sources..

Fig. S2 The optical transparency curves of the BN/PMMA composite film with different BN fractions.


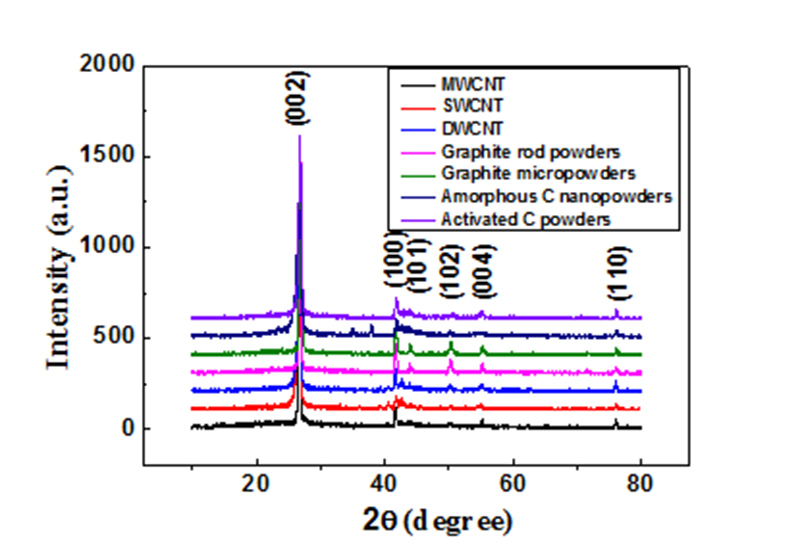


**A**


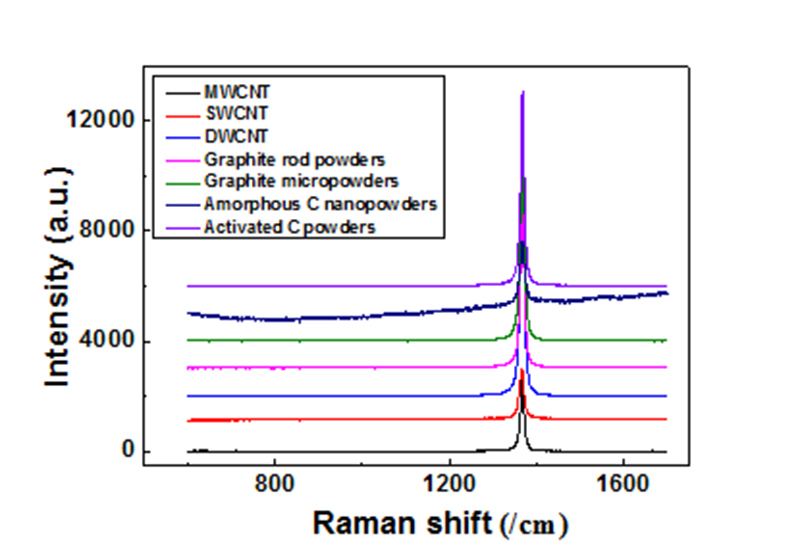


**B**

Figure S1

(Fei Liu *et al.* submitted to ***Scientific Reports***)


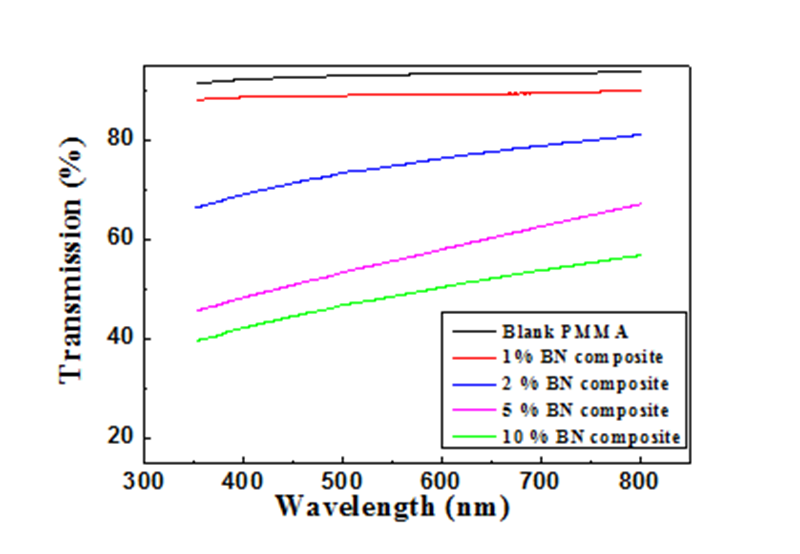


Figure S2

(Fei Liu *et al.* submitted to ***Scientific Reports***)

1. ^†^ [1, ^†^] F. Liu, X. S. Mo, H. B. Gan, T. Y. Guo, J. Chen, S. Z. Deng, N. S. Xu,

   State Key Laboratory of Optoelectronic Materials and Technologies, Guangdong Province Key Laboratory of Display Material and Technology, and School of Physics and Engineering, Sun Yat-sen University, Guangzhou 510275 (PR China),

   ^†^Corresponding Email: [liufei@mail.sysu.edu.cn](mailto:liufei@mail.sysu.edu.cn)

   [2] Y. Bando, F. Liu, Xuebin Wang, Inorganic Nanostructured Materials Group, MANA, National Institute for Materials Science, Namiki 1-1, Tsukuba, Ibaraki, Japan 305-0044,

   ^†^Corresponding Email: [Wangxb@fuji.waseda.jp](mailto:Wangxb@fuji.waseda.jp)

   [3] T. Sekiguchi, B. Chen, Nano-Electronics Materials Unit, MANA, National Institute for Materials Science, Namiki 1-1, Tsukuba, Ibaraki, Japan 305-0044

   [4] D. Golberg, Nanotube Group, MANA, National Institute for Materials Science, Namiki 1-1, Tsukuba, Ibaraki, Japan 305-0044 [↑](#footnote-ref-2)
